# Molecular Mechanism of Acrylamide Neurotoxicity: Lessons Learned from Organic Chemistry

**DOI:** 10.1289/ehp.1205432

**Published:** 2012-10-11

**Authors:** Richard M. LoPachin, Terrence Gavin

**Affiliations:** 1 Department of Anesthesiology, Montefiore Medical Center, Albert Einstein College of Medicine, Bronx, New York, USA; 2 Department of Chemistry, Iona College, New Rochelle, New York, USA

**Keywords:** HSAB theory, oxidative stress, protein adducts, soft electrophile, toxic axonopathy, type-2 alkenes, α,β-unsaturated carbonyl derivatives

## Abstract

Background: Acrylamide (ACR) produces cumulative neurotoxicity in exposed humans and laboratory animals through a direct inhibitory effect on presynaptic function.

Objectives: In this review, we delineate how knowledge of chemistry provided an unprecedented understanding of the ACR neurotoxic mechanism. We also show how application of the hard and soft, acids and bases (HSAB) theory led to the recognition that the α,β-unsaturated carbonyl structure of ACR is a soft electrophile that preferentially forms covalent bonds with soft nucleophiles.

Methods: *In vivo* proteomic and *in chemico* studies demonstrated that ACR formed covalent adducts with highly nucleophilic cysteine thiolate groups located within active sites of presynaptic proteins. Additional research showed that resulting protein inactivation disrupted nerve terminal processes and impaired neurotransmission.

Discussion: ACR is a type-2 alkene, a chemical class that includes structurally related electrophilic environmental pollutants (e.g., acrolein) and endogenous mediators of cellular oxidative stress (e.g., 4-hydroxy-2-nonenal). Members of this chemical family produce toxicity via a common molecular mechanism. Although individual environmental concentrations might not be toxicologically relevant, exposure to an ambient mixture of type-2 alkene pollutants could pose a significant risk to human health. Furthermore, environmentally derived type-2 alkenes might act synergistically with endogenously generated unsaturated aldehydes to amplify cellular damage and thereby accelerate human disease/injury processes that involve oxidative stress.

Conclusions: These possibilities have substantial implications for environmental risk assessment and were realized through an understanding of ACR adduct chemistry. The approach delineated here can be broadly applied because many toxicants of different chemical classes are electrophiles that produce toxicity by interacting with cellular proteins.

Acrylamide (ACR) is a water-soluble alkene used in the production of polymers and gels that have various commercial applications. For example, polyacrylamide preparations are used in the cosmetic, paper, and textile industries; in ore processing; and as soil conditioners and flocculants for wastewater treatment (Friedman 2003; Smith and Oehme 1991; Tilson 1979). Coincidental with the burgeoning industrial use of ACR monomer in the 1950s, it was quickly realized that cumulative neurotoxicity characterized by ataxia, skeletal muscle weakness, cognitive impairment, and numbness of the extremities was a potential outcome of occupational exposure (Deng et al. 1993; Garland and Patterson 1967; He et al. 1989; reviewed by Friedman 2003; Smith and Oehme, 1991; Spencer and Schaumburg 1974a; Tilson 1979). Early research involving laboratory animals showed that exposure to ACR monomer produced a neurotoxicity syndrome that resembled the neurological symptoms of human intoxication (reviewed by LoPachin and Lehning 1994; Spencer and Schaumburg 1974b; Tilson 1979). Morphological studies conducted during the late 1960s and early 1970s suggested that both human and experimental ACR neurotoxicities were associated with cerebellar Purkinje cell death and degeneration of distal axons and nerve terminals in the peripheral and central nervous systems (PNS and CNS, respectively) (reviewed by LoPachin 2004; LoPachin and Lehning 1994; LoPachin et al. 2003). In addition to characteristic neurotoxicity in adult humans and animals, there is more recent experimental evidence, albeit controversial, that prenatal and perinatal exposure of rodent pups to ACR causes neurodevelopmental toxicity (e.g., Friedman et al. 1999; Garey and Paule 2010; Takahashi et al. 2009).Whereas the majority of research indicates selective targeting of nervous tissue, rodent studies have also suggested that ACR causes reproductive toxicity [e.g., decreased litter size, DNA strand breaks (Tyl et al. 2000)] and an increased incidence of certain tumors [e.g., mammary gland fibroadenomas in female rats, tunica vaginalis mesotheliomas in male rats (Friedman et al. 1995; Johnson et al. 1986)]. However, to date, there is little evidence that these experimental non-neurotoxic consequences have human relevance (Haber et al. 2009; Mucci et al. 2003; Rice 2005).

Thus, the majority of evidence suggests that ACR exposure across broad daily dose-rates causes selective neurotoxicity in humans and laboratory animals. The early morphological descriptions of ACR neuropathy provided a framework for subsequent research that attempted to decipher the molecular mechanisms of neurotoxicity (reviewed by Friedman 2003; Howland 1985; Miller and Spencer 1985; LoPachin and Lehning 1994; Tilson 1979). Although many putative mechanisms and sites of ACR action were tested, for example, inhibition of Na^+^/K^+^-ATPase and the resulting reverse operation of the axolemmal Na^+^/Ca^2+^-exchanger (LoPachin and Lehning 1994), reduced fast axonal transport (Sickles et al. 2002) and inactivation of enzymes involved in neuronal energy production (Spencer et al. 1979), the identification of a necessary and sufficient neurotoxic process remained elusive. However, these early hypotheses were not developed within the framework that xenobiotics can produce toxicity by interacting directly with specific sites on cellular macromolecules (e.g., enzymes) and that this interaction is dictated by the chemical nature of the toxicant (Cohen et al. 1997; Coles 1984–1985; Hinson and Roberts 1992; see also Liebler 2008; LoPachin and DeCaprio 2005). Therefore, by understanding toxicant chemistry, plausible molecular-level sites and mechanisms of action can be predicted. In this review, we discuss the chemical nature of ACR (a soft electrophile) and how this determines the corresponding sites of protein adduction (soft nucleophilic sulfhydryl thiolates on cysteine residues). Basic recognition of the chemistry of toxicant–target reactions has led the development and testing of a rational mechanistic hypothesis of ACR neurotoxicity (see below). Although this review focuses on ACR, the proposed algorithm is broadly applicable to many different classes of chemical neurotoxicants, for example, heavy metals, quinones, and unsaturated aldehyde derivatives. In the following section, we provide a brief historical overview of ACR neurotoxicity in humans and laboratory animals.

## ACR Neurotoxicity: Evolving Neurobiological Concepts of the Distal Axonopathy

Daily exposure of laboratory animals (rodents, rabbits, primates, dogs, cats, and guinea pigs) to a broad range of ACR dose-rates (0.5–50 mg/kg/day) is associated with neurological deficits that resemble human neurotoxicity. Our early research was based on the contemporary concept that ACR produced central–peripheral distal axon degeneration and, accordingly, we focused on possible axonal sites of action [e.g., axolemmal Na^+^/K^+^-ATPase (Lehning et al. 1998; LoPachin et al. 1992, 1993, 2002b; reviewed by LoPachin and Lehning 1994)]. However, results from quantitative morphometric studies of the peripheral nerve suggested that axon degeneration was an epiphenomenon specifically related to lower ACR dose-rates (Lehning et al. 1998; LoPachin et al. 1992, 2000). Silver stain analyses of CNS tissue from ACR-intoxicated rats subsequently confirmed this dose-rate phenonmenon (Lehning et al. 2002a, 2002b, 2003; however, see Bowyer et al. 2009) and showed that regardless of exposure level, ACR intoxication was associated with selective nerve terminal degeneration in broad CNS regions. Therefore, these findings, in conjunction with data from earlier morphological, electrophysiological, and neurochemical studies (reviewed by LoPachin et al. 2002b) provided observational evidence that ACR disrupted neurotransmission. Accordingly, we proposed that nerve terminals were a primary site of ACR action and that neurotoxicity was a consequence of impaired synaptic transmission in the PNS and CNS (LoPachin 2004; LoPachin et al. 2003).

In formulating possible molecular mechanisms of presynaptic toxicity, we considered the fact that ACR was an electrophile that might produce neurotoxicity by binding to nucleophilic cysteine sites on proteins (Cavins and Friedman 1968; Friedman et al. 1965). In support of this possibility, it was recognized that the activities of many nerve terminal proteins were regulated by the ionization of specific cysteine sulfhydryl groups to highly reactive thiolates (Kiss 2000; Lipton et al. 2002; LoPachin and Barber 2006). We therefore hypothesized that ACR adduction of these regulatory residues might cause presynaptic toxicity, although some contemporary research did not support this idea (e.g., Martenson et al. 1995). Nonetheless, ensuing studies showed that ACR disrupted presynaptic neurotransmitter release, membrane re-uptake and vesicular storage by selectively forming adducts with cysteine residues on specific proteins involved in these processes, for example, *N*-ethylmaleimide (NEM)–sensitive factor (release), the dopamine membrane transporter (re-uptake) and the vesicular monoamine transporter (vesicular storage) (Barber and LoPachin 2004; Barber et al. 2007; LoPachin et al. 2004, 2006, 2007a, 2007b). Experimental evidence that ACR did not alter protein synthesis, energy production, or axonal transport indicated that presynaptic toxicity was a direct toxicant effect (reviewed by LoPachin and Lehning 1994). Whereas these data implied a central role for cysteine adduction in ACR neurotoxicity, it was not clear how such adduct formation might cause protein dysfunction and why nerve terminals were selectively vulnerable to the effects of protein adduction. This latter concern was particularly germane because most proteins contain at least one cysteine residue (Jones 2010) and ACR has been reported to form adducts with a variety of neuronal and non-neuronal proteins (e.g., Barber et al. 2007; LoPachin et al. 2004). As a consequence, it could not be assumed that adduct formation at a given cysteine residue had toxicological relevance. In the next section we discuss the adduct chemistry of ACR and show how this chemistry is related to the production of nerve terminal toxicity.

## ACR Adduct Chemistry: Covalent Interactions with Biological Nucleophiles

**Figure 1:**
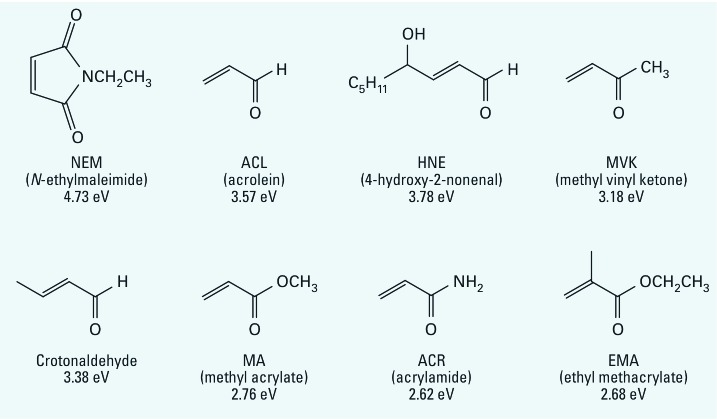
Line structures for several conjugated α,β-unsaturated carbonyl derivatives of the type-2 alkene chemical class. For each chemical, the electrophilic index (ω) is provided and the full chemical name is indicated in the parentheses.

ACR is a three-carbon α,β−unsaturated carbonyl derivative and is a member of a large chemical class known as type-2 alkenes (LoPachin et al. 2007a). Members of this class are characterized by a conjugated system formed when an electron-withdrawing group (e.g., the carbonyl group) is linked to an alkene carbon (Figure 1). The *pi* electrons in these conjugated systems are highly polarizable (mobile), and the carbonyl group of ACR withdraws electron density from the alkene to form an electron deficient (electrophilic) site at the β-carbon. As an electrophile, ACR, like many xenobiotic chemicals and/or their metabolites, causes cytotoxicity by forming covalent bonds with electron-rich (nucleophilic) residues on biological macromolecules (e.g., enzymes, DNA) (Hinson and Roberts 1992; LoPachin et al. 2012; Schwobel et al. 2011). Because ACR is an amide derivative, it does not undergo Schiff base formation with nucleophiles, but can form Michael-type adducts with nucleophiles via second-order addition reactions to the β-carbon. Electrophiles do not react arbitrarily with nucleophiles. Instead, these interactions exhibit a significant degree of selectivity as predicted by the hard and soft, acids and bases (HSAB) theory of Pearson (1990). Accordingly, electrophilic and nucleophilic molecules are classified as being either soft (relatively polarizable) or hard (relatively non-polarizable) and, based on this principle, toxic electrophiles will react selectively with biological targets of comparable softness or hardness. The unsaturated carbonyl structure of ACR is a soft electrophile that will preferentially form Michael-type adducts with soft nucleophiles, which in biological systems are sulfhydryl side-chains on cysteine residues. In contrast, although nitrogen groups on lysine (ε-amino groups) and histidine (imidazole ring) residues are also nucleophilic, these are harder sites and are therefore less favored targets for ACR adduction (see below).

The relative softness or hardness of an electrophile can be determined from the respective energies of the outermost or frontier molecular orbitals (FMOs). Because small molecule FMO energies can be calculated using various quantum mechanical models, HSAB parameters such as softness (σ) and hardness (η) of an electrophile are readily computed. With respect to covalent reactions, relative softness (σ) reflects the ease with which electron redistribution occurs during adduct (covalent bond) formation. Thus, the softer the electrophile (larger σ value), the faster it will accept electron density from a donating nucleophile. The values of σ and η also can be used in an algorithm to calculate the electrophilic index (ω) of a toxicant, the magnitude of which reflects the relative propensity of the electrophile to form an adduct with a given nucleophile (LoPachin et al. 2012; Schwobel et al. 2011). Indeed, substantial evidence suggests that σ and ω are determinants of the chemical reactions that mediate electrophile toxicity (LoPachin et al. 2012; Schultz et al. 2005, 2006; Schwobel et al. 2011). Whereas this is true for the majority of type-2 alkene electrophiles, physiochemical characteristics such as steric hindrance imposed by tertiary structure, solubility, and acid–base equilibrium can influence predictions of toxic potency based on HSAB parameters (reviewed by LoPachin et al. 2009a, 2009b, 2012). For example, Table 1 shows that when σ and ω values were calculated for a series of type-2 alkenes, the corresponding values were only qualitatively related to the second-order rate constants (*k*_2_) for type-2 alkene adduction of cysteine sulfhydryl groups and to the respective magnitudes of *in vitro* synaptosomal dysfunction [toxic potency or half maximal inhibitory concentration (IC_50_)] (LoPachin et al. 2007a, 2007b, 2009a, 2009b). This lack of correspondence is due to the slower than predicted reaction rate for 4-hydroxy-2-nonenal (HNE), that is, in the absence of HNE, the type-2 alkene σ values in Table 1 are closely correlated to the corresponding *k*_2_ values (*r*^2^ = 0.92; see LoPachin et al. 2007b). The slower adduct reaction is attributable to steric hindrance imposed by the bulky (–C_5_H_11_) alkyl tail of HNE (Friedman and Wall 1966). Such discrepancies are expected because the HSAB algorithms incorporate electronic components but not three-dimensional features of chemical structure that can influence the toxicological outcome. Nonetheless, it is evident that ACR is a relatively weak electrophile (low ω value) that slowly forms adducts with cysteine residues (slow second-order reaction rate; Table 1).

**Table 1:** Calculated HSAB and experimental parameters for conjugated type-2 alkenes and ­nonconjugated analogs.

Type-2 alkene	σ (× 10–3/eV)a	ω (eV)	log k2b	Uptake (log IC50)c
Acrolein	379	3.57	2.596	–4.28
NEM	406	4.73	6.536	–4.33
MVK	382	3.18	2.048	–3.48
HNE	393	3.78	0.938	–3.40
Crotonaldehyde	385	3.38	ND	ND
MA	315	2.76	–1.893	–0.34
ACR	346	2.62	–1.804	–0.36
EMA	322	2.68	ND	ND
Nonconjugatedd
Propanal	323	2.26	—	—
Allyl alcohol	276	1.63	—	—
Abbreviations: EMA, ethyl methacrylate; MA, methyl acrylate; MVK, methylvinyl ketone; ND, not determined. aFor each compound, respective lowest and highest occupied molecular orbital energies (ELUMO and EHOMO, respectively) were obtained from ground state equilibrium geometries with density functional theory calculations DFT B3LYP-6-31G* in vacuum from 6-31G* initial geometries and were used to calculate softness (σ) and the electrophilic index (ω) as described by LoPachin et al. (2012). bSecond-order reaction rates (k2) were determined for type-2 alkene reactions with l-cysteine at pH 7.4. cInhibition of synaptosomal membrane tritiated dopamine [(3H)-DA] uptake was determined in striatal synaptosomes exposed to type-2 alkenes (LoPachin et al. 2007a, 2007b). dDo not undergo the Michael reaction.								

The weak electrophilic character of ACR seems inconsistent with the well-documented ability of this chemical to cause significant neurotoxicity. However, the second-order reaction rate for the formation of ACR-cysteine adducts is governed not only by the relative concentrations of each reactant but also by the electrophilicity of the electron acceptor (ACR, see above) and the relative nucleophilicity of the electron donor (cysteine sulfhydryl group). Thus, the nucleophilic strength of the sulfhydryl target can affect the energy of the transition state for adduct formation and hence the magnitude of the corresponding rate constant (*k*_2_). As indicated above, soft electrophiles such as ACR preferentially react with soft nucleophiles. The softness of a nucleophile reflects its relative ability to rapidly transfer electron density to the electrophile. In aqueous environments, sulfhydryl groups on cysteine residues exist in a pH-dependent equilibrium that determines the respective concentrations of the protonated thiol (RSH) and non-protonated thiolate (RS^–^) forms. Corresponding calculations of nucleophilic softness (σ; Table 2) indicate that the thiolate is substantially softer than the thiol. The side chain nitrogen nucleophiles of histidine and lysine residues, as well as the protonated ε-amino group nitrogen of lysine, are also harder moieties than the sulfhydryl thiolate (Table 2). Based on the HSAB premise of soft–soft interactions, these data identify the sulfhydryl thiolate state of cysteine residues as the preferred target of ACR. The extent to which a given nucleophile will react with ACR can be predicted by calculating the nucleophilicity index (ω^–^). This HSAB-derived parameter utilizes the hardness (η) and chemical potential (μ) of both ACR (electrophile) and possible nucleophilic amino acid targets (LoPachin et al. 2008a, 2012). The significantly lower ω^–^ values for the harder nucleophiles (Table 2) indicate that ACR targets soft cysteine thiolate sites. This type of calculation also demonstrates that, relative to ACR, acrolein is a softer and more electrophilic type-2 alkene that reacts much faster with sulfhydryl thiolates (Table 2). The thiolate predilection of ACR and other type-2 alkenes based on HSAB calculations has been experimentally confirmed using proteomic and *in chemico* approaches (Cavins and Friedman 1968; Friedman et al. 1965; LoPachin et al. 2007a, 2007b, 2009a; Martyniuk et al. 2011).

**Table 2:** Interactions with type-2 alkenes with potential amino acid target: calculated HSAB parameters.

Residue	Side chain group	σ × 10–3/eV	ACR ω– × 10–3 eV (relative)	Acrolein ω– × 10–3 eV (relative)
CYS (–1)	–CH2S–	382	146 (1.00)	266 (1.00)
LYS (0)	–(CH2)4NH2	285	56.6 (0.39)	126 (0.47)
HIS (0)	313	48.5 (0.33)	114 (0.43)
CYS (0)	–CH2SH	282	40.0 (0.27)	98.4 (0.37)
LYS (+1)	–(CH2)4NH3+	271	35.3 (0.24)	90.0 (0.34)
For each amino acid nucleophile, HSAB parameters were calculated on the basis of selected ionization states (in parentheses). Data show that the sulfhydryl thiolate state is a significantly softer (σ) nucleophile than either the corresponding thiol state or the other amino acid residues such as histidine or lysine. This characteristic indicates that the thiolate state will react selectively with comparably soft electrophiles such as acrolein. The nucleophilic index (ω–), which reflects the propensity of adduct formation, indicates that the sulfhydryl thiolate state is the preferential target of the type-2 alkenes. Relative to the thiolate state (1.00), thiol groups and the lysine and histidine residues are substantially less competitive targets for type-2 alkene adduct formation (mean relative value, 0.35).								

## Catalytic Triads as the Molecular Sites of ACR Action

Both *in vivo* and *in vitro* proteomic studies (e.g., Barber and LoPachin 2004; Cai et al. 2009; Doorn and Petersen 2003; reviewed by LoPachin et al. 2012) have indicated that ACR and other type-2 alkenes impair protein function by reacting with specific cysteine residues on cellular proteins. For example, ACR inhibits presynaptic Na^+^-dependent dopamine transporter function by reacting with Cys342 (Barber et al. 2007); NEM forms adducts with Cys254 and thereby inhibits presynaptic vesicle (H^+^)-ATPase activity (Barber et al. 2007; Feng and Forgac 1992); and HNE adduct formation at Cys280 inhibits mitochondrial SIRT3 (SIRT3) activity (Fritz et al. 2011). However, it is unclear why these specific residues were targeted and, because the functional importance of these cysteines is not known, the toxicological relevance of this adduct formation is uncertain. The preceding discussion suggests that such targeting might reflect the interaction of these type-2 alkenes with the highly nucleophilic sulfhydryl thiolate state of cysteine residues. However, the p*K*_a_ of the sulfhydryl side chain is approximately 8.4 and therefore, at intracellular pH ranges (7.0–7.4), these groups exist mostly in the non-nucleophilic thiol state (Table 2). Nonetheless, sulfhydryl thiolate groups can be found in cysteine-centered catalytic triads and other microenvironments that significantly reduce side chain p*K*_a_ values. The ionization of these sulfhydryl groups, and therefore the corresponding nucleophilicity, is determined by proton shuttling that occurs among basic (histidine, arginine, lysine) and acidic (aspartate, glutamate) amino acid residues that are brought into proximity via the tertiary structure of the protein, for example, the arginine_357_-cysteine_121_-aspartate_355_ motif of methionine adenosyl-transferase (Gutteridge and Thornton 2005; LoPachin and Barber 2006). Thus, although the majority of sulfhydryl groups in proteins exist primarily (> 90%) in the nonreactive thiol state; those present in catalytic triads are ionized to a much greater extent and, consequently, will react significantly faster with electrophiles. This concept is exemplified by the ryanodine-responsive calcium-release channel of skeletal muscle, where 1 of 50 cysteine residues is reactive because of its presence in a catalytic triad (Sun et al. 2001). Cysteine catalytic triads are often located within the active sites of many critical nerve terminal enzymes (e.g., NEM-sensitive factor, vesicular monoamine transporter). The highly nucleophilic sulfhydryl thiolate sites regulate enzyme activity by acting as acceptors for redox modulators such as nitric oxide (NO) or by playing a direct role in corresponding catalytic activity (reviewed by Jones 2010; LoPachin and Barber 2006; Winterbourn and Hampton 2008). Thus, it should be evident that adduction of the triad sulfhydryl thiolate will have substantial implications for protein function and subsequent presynaptic toxicity (see below).

To investigate the possibility that ACR targeted cysteine residues in catalytic triads, we (Martyniuk et al. 2011) determined the effects of selected type-2 alkenes on the activity of recombinant human erythrocyte glyceraldehyde-3-phosphate dehydrogenase (GAPDH), which contains a regulatory cysteine-centered (Cys152) catalytic triad (Thomas et al. 1995). Consistent with HSAB concepts, the softness (σ) and electrophilicity (ω) values for ACR and the other type-2 alkenes tested [acrolein, methylvinyl ketone (MVK)] were related to the corresponding second-order rate constants (log *k*_2_) and potencies (log *K*_I_) for GAPDH inhibition (Table 3). Tandem mass spectrometry was used to quantify the adduct formation associated with graded concentrations of ACR. Results indicated that lower *in vitro* concentrations of ACR inhibited GAPDH activity by selectively forming adducts with Cys152 in the active site of this enzyme, whereas at higher concentrations ACR also reacted with Cys156 and Cys247. Calculations using the PROPKA program (Jensen Research Group, Copenhagen, Denmark) revealed a p*K*_a_ of 6.03 for Cys152, whereas the p*K*_a_ values for Cys156 and Cys247 were higher. Furthermore, we found that GAPDH inhibition by the selected type-2 alkenes was pH-dependent, which also indicated thiolate mediation. These data suggest that Cys152 of GAPDH exists in a p*K*_a_-lowering microenvironment and that ACR inhibited enzyme function by preferentially forming irreversible Michael-type adducts with this highly nucleophilic sulfhydryl thiolate site. In general, cysteine thiolates contained within catalytic triads function as acceptors for electrophilic mediators of redox signaling [e.g., NO, hydrogen peroxide (H_2_O_2_)] and, therefore, ACR adduction of these sites might impair protein function by disrupting this neuromodulatory signaling (LoPachin et al. 2008b, 2009a, 2009b).

**Table 3:** Type-2 alkene HSAB and kinetic parameters for interactions with GAPDH.

Electrophilea	σ (× 10–3/eV)	ω (eV)	log k2	log KI
Acroleinb	371	3.82	4.250	–4.419
MVK	363	3.38	3.885	–4.220
ACR	315	2.61	0.502	–0.607
aHSAB (σ, ω) and kinetic parameters (k2, KI) were calculated as described by Martyniuk et al. (2012). bBased on the HSAB parameters, acrolein and MVK are significantly softer and more reactive electrophiles than ACR (i.e., larger values of σ and ω, respectively). The rank orders of respective σ and ω values for each type-2 alkene were closely correlated to the corresponding rate constants (k2; r2 = 0.9996 and 0.9359, respectively) and relative potencies (KI; r2 = 0.9926 and 0.9004, respectively) for inhibition of GAPDH activity.								

## Molecular Mechanism of ACR Synaptotoxicity

The preceding discussion suggests that ACR and other type-2 alkenes preferentially form irreversible adducts with sulfhydryl thiolate groups that also function as acceptors for NO and other redox neuromodulators, for example, the thiolate of Cys152 on GAPDH is an NO acceptor (Mohr et al. 1994). NO is a biological electrophile that forms reversible adducts with sulfhydryl thiolate groups (*S*-nitrosylation) on proteins. NO signaling transiently decreases synaptic strength by reversibly inhibiting the function of several proteins involved in the synaptic vesicle cycle, for example, NEM-sensitive factor (release), the dopamine membrane transporter (re-uptake) and the vesicular monoamine transporter (vesicular storage) (Kiss 2000; LoPachin and Barber 2006; Rudkouskaya et al. 2010). It is highly significant that NO and ACR have similar inhibitory effects on protein function and that the NO-sensitive proteome exhibits substantial overlap with the ACR-adducted proteome (Barber and LoPachin 2004; Barber et al. 2007; LoPachin et al. 2004; Martyniuk et al. 2011). This correspondence suggests that ACR mimics the protein effects of the redox neuromodulators (inactivation) by reacting with thiolate acceptors in catalytic triads. In contrast, the resulting irreversible blockade of redox signaling causes loss of NO-directed neuromodulation and ensuing synaptic toxicity.

NO signaling, however, is characteristic of most cell types (Hess et al. 2005) and ACR will form adducts with the thiolate acceptor sites of these non-neuronal cells (Barber et al. 2007). The proposed NO-based mechanism of ACR-induced synaptotoxicity therefore lacks nerve terminal specificity. Nonetheless, several unique anatomical and functional characteristics predispose this neuronal region to cumulative electrophilic attack. Specifically, neurotransmission is a complex multistep process that is highly regulated by NO signaling (reviewed by LoPachin and Barber 2006) and, therefore, ACR disruption of this pathway is likely to have significant consequences for presynaptic function. Furthermore, because the nerve terminal is anatomically separated from the cell body, it is devoid of transcriptional or translational capacity. Thus, unlike the cell body, the nerve terminal lacks the ability to initiate transcription-based reparative or cytoprotective responses, for example, the Nrf2-Keap1 antioxidant response (Zhang et al. 2011). In the absence of machinery for protein synthesis, maintenance of the presynaptic proteome is dependent on cell body protein manufacturing and subsequent anterograde axonal transport. Correspondingly, as a mechanism to limit material expenditure and increase efficiency, the turnover rates of many nerve terminal proteins are exceptionally slow relative to those of proteins in the nerve cell body or other cell types (Barber and LoPachin 2004; Barber et al. 2007; Calakos and Scheller 1996; Katyare and Shallom 1988; Lin and Scheller 2000). Thus, presynaptic proteins inactivated by cysteine adduct formation will be replaced slowly and will consequently accumulate as the rate of adduct formation exceeds the rate of removal by protein turnover. In contrast, dysfunctional adduct-inactivated proteins with short half-lives will not accumulate because they are rapidly replaced by the turnover process. Indeed, our proteomic studies have demonstrated a presynaptic buildup of cysteine adducts that is progressive and closely correlated to the development of ACR neurological symptoms. Furthermore, we have provided evidence that CNS nerve terminal dysfunction occurs at a cumulative adduct level of 350–500 pg cysteine adduct/μg protein. This reflects the minimal exposure threshold below which neurotoxicity does not occur because this level of adduct formation (i.e., < 350 pg cysteine adduct/μg protein) does not affect synaptic processes (Barber and LoPachin 2004; Barber et al. 2007; LoPachin et al. 2004). As intoxication continues and adduct formation exceeds this threshold, the pool of dysfunctional proteins increases proportionately and the related presynaptic processes are progressively disabled, leading to the characteristic cumulative neurotoxicity of ACR (LoPachin et al. 2002b, 2004, 2006).

The preceding discussion indicates that several anatomical and neurophysiological attributes render nerve terminals selectively vulnerable to dysfunction via cumulative electrophilic attack. However, as mentioned above, ACR is a type-2 alkene and therefore shares a common mechanism of toxicity with other structurally related members of this chemical class (e.g., acrolein, MVK, NEM). Although selective neurotoxicity is a clearly defined outcome in ACR-exposed human cohorts, similar exposure to other members of this class is associated with systemic toxicity, that is, cardiovascular, respiratory, hepatic or renal toxicity (Bisesi 1994; Tucek et al. 2002). This diversity of toxic responses is not related to mechanistic differences among members of this chemical family, rather it is due to variations in electrophilic reactivity that correspondingly influence toxicokinetics and tissue distribution (Gillette et al. 1974; Rozman and Klaassen 2001). Thus, highly electrophilic type-2 alkenes such as acrolein (Table 1) rapidly form adducts with protein sulfhydryl thiolate groups at systemic sites of absorption. Adduct formation is not only the mechanism of acrolein toxicity, but also restricts the corresponding tissue distribution. As a consequence of this restriction, the toxic manifestations of acrolein and other reactive electrophiles are characteristic of the absorption site; for example, acrolein inhalation produces pulmonary toxicity, whereas systemic administration is associated with hepatic or vascular toxicity (Green and Egle 1983; Parent et al. 1996; Struve et al. 2008). In contrast, as a weak water-soluble electrophile, ACR slowly forms thiolate adducts and is therefore less susceptible to the limiting influence of systemic “adduct buffering.” Accordingly, ACR has a large volume of distribution and readily crosses the blood-brain barrier (Barber et al. 2001). Based on theoretically similar CNS accessibilities, systemic exposure to methyl acrylate, ethyl methacrylate, or other weak type-2 alkene electrophiles (Table 1) should also cause selective neurotoxicity. Indeed, the results of both human (Sadoh et al. 1999; Seppalainen and Rajaniemi 1984) and animal (Abou-Donia et al. 2000) studies suggest that exposure to these chemicals can produce ACR-like neurotoxicity. Clearly, relative softness (σ) and electrophilicity (ω) determine not only the toxicodynamic character of ACR and other type-2 alkenes (i.e., amino acid targets and mechanisms of toxicity), but also tissue distribution and corresponding toxic manifestations.

The preceding discussion indicates that the most toxicologically relevant targets of ACR are those nerve terminal proteins that turnover slowly and are importantly involved in neurotransmitter release, storage, and re-uptake. Whereas the adduct chemistry of ACR has been considered through the perspective of nerve terminal damage, future research might confirm an alternative site of neuronal (or glial) action. Regardless of the identified site, a confluence of evidence stemming from early *in chemico* studies (e.g., Cavins and Friedman 1968) to recent proteomic research (e.g., LoPachin et al. 2007b) suggests that the mechanism of toxicity will involve the soft–soft covalent interactions of ACR with cysteine thiolate groups.

## Possible Environmental Significance of ACR and Other Type-2 Alkenes

As stated above, ACR is used to manufacture polymers that have broad commercial, industrial, and agricultural applications and, therefore, occupational or accidental intoxication was considered historically to be the primary cause of acquired neurotoxicity. However, other sources of significant daily ACR exposure are now recognized, that is, air/water pollution, cigarette smoke, and diet (Friedman 2003; Perez et al. 1999; Smith et al. 2000; Tornqvist 2005; Tucek et al. 2002). Although it has been estimated that the human body burden from these sources can be up to 30 μg ACR/kg/day (Food and Agriculture Organization of the United Nations and the World Health Organization 2005), the neurotoxicological significance of this exposure level is questionable (Boon et al. 2005; Hagmar et al. 2005; Kutting et al. 2009). However, as indicated above, ACR is a member of the type-2 alkene chemical class, which is a large group of structurally related compounds used extensively in the manufacturing, agricultural, polymer, and pharmaceutical industries. As a result, human exposure to the type-2 alkenes is ubiquitous and potentially harmful because many of these compounds are well-documented toxicants. Specifically, unsaturated aldehydes and carbonyls (acrolein, acrylonitrile, MVK) are significant components of air pollution, automobile exhaust, and smoke from cigarette, wood, and coal combustion (Andrews and Clary 1986; Bisesi 1994; Faroon et al. 2008; Feron et al. 1991; Fujioka and Shibamoto 2006; Stevens and Maier 2008; Tucek et al. 2002; Woodruff et al. 2007). At least 36 different unsaturated aldehydes (mostly type-2 alkenes) have been found in the U.S. water supply, often at levels exceeding maximal recommended concentrations. In fact, with the exception of heavy metals, aldehdyes are considered to be the major contaminants in drinking water (reviewed by Andrews and Clary 1986; Bisesi 1994; Conklin et al. 2010; Faroon et al. 2008; Feron et al. 1991; Tucek et al. 2002). There is experimental evidence that the toxic consequences of environmental exposure are mediated by type-2 alkenes (Andre et al. 2008; Danielsen et al. 2011; Facchinetti et al. 2007; Moretto et al. 2009). Finally, over 300 type-2 alkenes are natural constituents (e.g., acrolein, crotonaldehyde) of various foods and additional carbonyl, aldehyde, and ketone derivatives are produced during cooking fats, oils, and sugars. Based on dietary consumption alone, it is estimated that the α,β-unsaturated aldehyde burden in humans is nearly 200 μg/kg-body wt/day (Conklin et al. 2010; Wang et al. 2008).

Human populations are therefore exposed to complex type-2 alkene mixtures, the chemical composition and corresponding concentrations of which depend on several variables including geographical location, personal habits (diet, tobacco usage), and occupation (Bisesi 1994; Faroon et al. 2008; Feron et al. 1991; Friedman 2003; Stevens and Maier 2008; Tucek et al. 2002; Woodruff et al. 2007). Of particular concern, research has shown that these environmental toxicants produce cell damage via a common molecular mechanism, that is, protein dysfunction through formation of Michael-type adducts with sulfhydryl groups on specific cysteine residues (e.g., Dalle-Donne et al. 2007; Doorn and Petersen 2003; LoPachin et al. 2007a, 2007b, 2009a, 2009b; Nerland et al. 2003; Martyniuk et al. 2011). Thus, although the environmental concentrations of any particular unsaturated compound might not be sufficient to cause toxicity, continuous low-level exposure to a mixture of type-2 alkenes might be toxicologically significant (Kamel and Hoppin 2004; LoPachin et al. 2008a, 2008b, 2009b).

In addition to the environmental prevalence of the type-2 alkenes, acrolein, HNE, 4-oxy-2-nonenal (ONE), and other members of this chemical class are produced endogenously during membrane lipid peroxidation associated with cellular oxidative stress. There is growing evidence that these endogenous type-2 alkenes play a pathogenic role in disease processes and traumatic tissue injuries that have oxidative stress as a molecular etiology, for example, stroke, atherosclerosis, Alzheimer’s disease, spinal cord trauma, and diabetes (Butterfield et al. 2010; Grimsrud et al. 2008; Hamann et al. 2008; Uchida 2003; Zarkovic 2003). Therefore, based on their common toxic mechanism, environmentally derived type-2 alkenes might act either synergistically or additively with endogenously generated unsaturated aldehydes. This interaction could amplify the extent of cellular damage and thereby accelerate development of the disease/injury process. That this idea has toxicological plausibility is suggested by epidemiological and experimental research indicating an association between environmental toxicant exposure (e.g., pesticides, heavy metals, industrial chemicals) and an increase in the incidence and severity of many human diseases (Brown et al. 2005, 2006; Grandjean and Landrigan 2006; Kamel and Hoppin 2004; Landrigan et al. 2005; O’Toole et al. 2008). With specific reference to environmental type-2 alkene exposure, research has shown that dietary consumption of acrolein exacerbates myocardial ischemic injury and atherosclerosis in mice by interacting with endogenous unsaturated aldehydes generated during ongoing oxidative stress (Conklin et al. 2010; Ismahil et al. 2011; Luo et al. 2007; Srivastava et al. 2011; Wang et al. 2008). On the basis of these studies it has been proposed that chronic environmental exposure to unsaturated aldehydes is a significant risk factor for cardiovascular diseases (Luo et al. 2007; O’Toole et al. 2008; Wang et al. 2008). Similarly, we have suggested that environmental exposure to a mixture of weak type-2 alkene electrophiles (e.g., ACR, methyl acrylate, ethyl methacrylate) could accelerate the progressive nerve terminal demise associated with Alzheimer’s disease (reviewed by LoPachin et al. 2008b, 2009b). In support of this, there is now considerable evidence that the Alzheimer’s disease pathogenic mechanism involves neuronal oxidative stress with subsequent generation of highly reactive type-2 alkene derivatives including acrolein, HNE, and ONE (Ansari and Scheff 2010; Butterfield et al. 2010; Nam et al. 2010; Singh et al. 2010; Sultana and Butterfield 2010). Furthermore, evidence suggests that nerve terminal dysfunction in relevant brain regions precedes neurodegeneration and is a primary pathophysiological event in Alzheimer’s disease (reviewed by Coleman et al. 2004; Forero et al. 2006; Keating 2008; LoPachin et al. 2008a; Selkoe 2002). Thus, presynaptic dysfunction in Alzheimer’s disease could be mediated by both environmental and endogenous type-2 alkenes (e.g., Keller et al. 1997; LoPachin et al. 2007a, 2007b, 2009a; Morel et al. 2002; Pocernich et al. 2001). Along these lines, subchronic human exposures to environmental matrices that contain significant type-2 alkene concentrations such as air pollution (Calderón-Garcidueñas et al. 2011; Chen and Schwartz 2009; Levesque et al. 2011) or cigarette smoke (Fujioka and Shibamoto 2006; Smith et al. 2000; Werley et al. 2008) are associated with an increased incidence of neurodegenerative conditions (e.g., Almeida et al. 2008; Cataldo et al. 2010; Chen and Schwartz 2009; Juan et al. 2004; Levesque et al. 2011; Peters et al. 2008; Tucek et al. 2002). Whereas other toxicant classes in these complex matrices could contribute to the corresponding neuropathogenic processes, the type-2 alkenes are distinguished by their exogenous prevalence, their common toxic mechanism, and their endogenous role in oxidative stress (see above). Despite this growing evidence, the potential for toxic synergy among members of the type-2 alkene class has largely gone unrecognized. As a result, risk assessment has been based on analyses of individual unsaturated carbonyls and their respective toxicities. However, from both a research and risk management perspective, future toxicological considerations should include the interactive potential of these chemicals.

## Summary

Early studies of ACR neurotoxicity involved observational research designed to define cell-level sites of action, for example, axon versus nerve terminal. Subsequent research was directed toward determining corresponding molecular mechanisms and, accordingly, numerous mechanistic scenarios were proposed and subsequently tested. Nonetheless, whether the selected neurophysiological parameter tested was a rational and therefore toxicologically plausible target could not be determined because significant mechanistic ambiguity existed at the chemical and molecular levels. However, mechanistic investigations were significantly advanced by recognizing the specific electrophilic nature of ACR and understanding the implications of this electronic character on the selective nucleophile targeting that determines the corresponding covalent adduct chemistry. Thus, we realized that ACR was a soft electrophile that preferentially formed adducts with soft nucleophilic sites on macromolecules. This pointed to the soft, highly nucleophilic thiolate states of cysteine residues in protein catalytic triads as toxicologically relevant molecular targets. Because thiolate sulfhydryl groups on proteins acted as regulatory acceptors for electrophilic mediators of redox signaling (e.g., NO), we ultimately provided evidence that ACR reduced neurotransmission at central and peripheral synapses by disrupting these signaling pathways. Also critical was the recognition that the relevant electronic characteristics defining the chemical basis for ACR toxicity were shared by other α,β-unsaturated carbonyl derivatives and possibly the entire type-2 alkene chemical class. This is a potentially significant realization because the type-2 alkenes are a unique group of structurally related unsaturated carbonyl, aldehyde, and ketone derivatives that are well-documented environmental toxicants and/or endogenous mediators of disease/injury processes associated with cellular oxidative stress. Based on their demonstrated common mechanism of toxicity, we propose that environmental exposure to a mixture of type-2 alkenes could represent a significant human health risk. Furthermore, these exogenously derived toxicants could interact synergistically with endogenous unsaturated aldehydes and thereby accelerate the onset and development of atherosclerosis, diabetes, Alzheimer’s disease, and other pathogenic conditions that have cellular oxidative stress as a molecular etiology. Thus, in this review we have described a relatively detailed mechanistic scenario for ACR neurotoxicity. This level of comprehension was achieved through understanding the principles of organic chemistry that govern the covalent interactions of electrophilic toxicants with their nucleophilic targets. Because many toxicants are electrophiles of varying softness and reactivity (e.g., methylmercury; *N*-acetyl-p-benzoquinone imine, 2,5-hexanedione) a similar approach could be used to identify rational nucleophilic targets on biological macromolecules.
